# The Treatment of Tuberculosis by Means of the Immune Substances (I.K.) Therapy

**Published:** 1912-12

**Authors:** 


### Reviews of Books.

**The Treatment of Tuberculosis by means of the Immune Substances (I.K.) Therapy.** An Introduction to CARL SPENGLER'S work on Immunity. By WALTER H. FEARIS, M.D. Pp. xx, 206. London: John Murray. 1912. Price, 6s. net.

This small volume is an outline of that system of "vaccine" treatment now advocated by Spengler, of Davos, and we believe that it is a handy and eminently practical abridgment of a larger work that is to follow and contain the more complete data upon which this form of treatment is based.

The work of Spengler is less generally familiar in this country than it is on the continent. Perhaps the rapidity with which he has introduced new methods of treatment by inoculation, each claimed to be a great advance upon the previous great improvement, has done something to discount his work with us; but in fairness to him it must be frankly admitted that he makes no statement which he is unprepared to support with experimental or clinical data, and his output of valuable and brilliant research is colossal.

Soon after the discovery of tuberculin Spengler began to experiment with it, and during the days of its greatest unpopularity he staunchly supported its claims. He introduced bovine tuberculin, and the alternating use of bovine and human tuberculin.

Spengler uses the term " vaccine " differently from our use of it. With us it means a suspension of bacteria, whereas he uses it to mean " the toxins which in a particular case cause little toxic-febrile reaction, although they induce marked local reaction and generation of immune substances," and these toxins are " derived from the non-predominant type of tubercle bacillus."

His preparation for the treatment of tuberculosis is made from the red blood-cells of an animal immunised by the use of a living culture of virulent tubercle bacilli, of the " Koch's brevis and the Humano-longus types," and also other bacteria frequently found in association with tubercle bacilli. It is called I.K., from the compound word " Immunkörper " (" immune bodies "). The chief immune bodies in I.K. are the bacteriolysins and antitoxins of tubercle bacilli, and of the complicating organisms most commonly associated with tuberculosis of the lungs. Hence two advantages over the use of tuberculin at once appear: firstly, that with I.K. a mixed infection is treated by the immune bodies of tubercle, and also of the various common organisms of a mixed infection; and secondly that the antitoxins contained *ready formed* in the I.K. are usually in sufficient quantity to neutralise the toxins liberated from the bacilli killed by the bacteriolysins of I.K., toxins so often liberated in a dangerously excessive amount when tuberculin is carelessly used, and sometimes even in spite of the greatest care in peculiarly sensitive patients.

Spengler states that should a strong reaction occur as the result of an inoculation of I.K., it is easily controlled by giving a far smaller dose. It would be a valuable help if it similarly control a reaction due to tuberculin.

It is claimed that the I.K. treatment is more effective and more rapid in producing its effect than tuberculin, that it is less dangerous than tuberculin, that it is the treatment best adapted for Sanatoria, and for the " domiciliary treatment " that is to be such a great factor in the tuberculosis campaign, and also that it is cheap.

Judging from past experience with tuberculin, there is a very real danger of this treatment being condemned solely because it will be used with a culpable disregard of the rules and directions that have been laid down for its use. I.K. treatment differs widely from the tuberculin treatment, and the directions for use by Spengler and others are set forth clearly in this little book, and no one should attempt this treatment who has not this book in his possession, or else has ready access to the German literature on Spengler's work.

Without attempting to express an opinion on the therapeutic value of I.K., we feel bound to say that Mr. Walter H. Fearis, in dealing with an intricate and complicated subject, has produced a book that is remarkably easy to read and clear to understand. Even those who have hardly glanced at recent work on immunity will find little difficulty in digesting this work, and those well versed in the practical use of tuberculin will gain a clearer understanding of the theories upon which their practice should be based, and will gain greater confidence in its use. Those who already are in possession of the original works and translations of Dr. Egbert Morland of Arosa, on the use of tuberculin, in our opinion the most generally useful and reliable books on that form of treatment, should add this volume to their bookshelves also.

In a book so well written, the book of a teacher, it seems a shame to draw attention to small faults ; but the author shows, here and there, some difficulty in translating German medical terms into their English equivalents, so that when one reads "Broncholoquy" it is impossible, unless one knows, to say whether it is something different from "Bronchophony," or a very questionable attempt at an improvement on the latter word ; and in using upper-case type on page 23 we should prefer "KOERNER" to the form "KORNER," for what would be in lower-case "Körner."

The type, paper, and especially the binding, are peculiarly pleasing and attractive.

